# Comparisons among Machine Learning Models for the Prediction of Hypercholestrolemia Associated with Exposure to Lead, Mercury, and Cadmium

**DOI:** 10.3390/ijerph16152666

**Published:** 2019-07-25

**Authors:** Hyejin Park, Kisok Kim

**Affiliations:** 1 Department of International Healthcare Administration, Daegu Catholic University, Gyeongsan 38430, Korea; 2 College of Pharmacy, Keimyung University, Daegu 42601, Korea

**Keywords:** machine learning, predictive model, heavy metals, cholesterol

## Abstract

Lead, mercury, and cadmium are common environmental pollutants in industrialized countries, but their combined impact on hypercholesterolemia (HC) is poorly understood. The aim of this study was to compare the performance of various machine learning (ML) models to predict the prevalence of HC associated with exposure to lead, mercury, and cadmium. A total of 10,089 participants of the Korea National Health and Nutrition Examination Surveys 2008–2013 were selected and their demographic characteristics, blood concentration of metals, and total cholesterol levels were collected for analysis. For prediction, five ML models, including logistic regression (LR), k-nearest neighbors, decision trees, random forests, and support vector machines (SVM) were constructed and their predictive performances were compared. Of the five ML models, the SVM model was the most accurate and the LR model had the highest area under receiver operating characteristic (ROC) curve of 0.718 (95% CI: 0.688–0.748). This study shows the potential of various ML methods to predict HC associated with exposure to metals using population-based survey data.

## 1. Introduction

People can be exposed to high levels of toxic metals from numerous sources, including contaminated air, water, soil, and food [1]. Among toxic metals, lead (Pb), mercury (Hg), and cadmium (Cd) are widely dispersed throughout the environment and can be detected in the blood, serum, and urine of people living in polluted areas [2]. Although the general population is often exposed to these metals simultaneously, most studies concerning the health effects of these metals have been carried out on animals, or on human populations with relatively high levels of exposure to individual metals [3,4,5]. Besides, recent studies show that toxic metals interact with other metals in various tissues [6,7]. However, few epidemiological studies have addressed the biological effects of low levels of exposure to mixtures of these metals, particularly with regard to possible interactions between metals.

Exposure to lead, mercury, or cadmium is known to cause various toxic effects and diseases, including disturbance of lipid metabolism or dyslipidemia [8]. There are many experimental and epidemiological studies showing that exposure to lead alters the concentration of total cholesterol in serum [9,10,11] and a strong positive association between blood concentration of mercury and serum total cholesterol level [12,13,14]. Furthermore, several animal studies have shown that exposure to cadmium significantly increases the serum levels of total cholesterol [15,16,17]. However, we lack information on the effect of combined exposure to these metal mixtures on cholesterol metabolism in the general population.

Recently, machine learning (ML) prediction models, such as logistic regression (LR) models, k-nearest neighbor (KNN), decision trees (DT), random forests (RF), and support vector machines (SVM) have been developed in many areas of health care research [18,19,20]. ML allows intelligent systems to build appropriate prediction models and is increasingly used to develop algorithms that classify individuals with complex interaction factors [21]. Our hypothesis is that ML analysis of survey data including body burden of metals can be used to identify individuals at high risk of hypercholesterolemia (HC). Therefore, in this study, we aimed to construct several ML models to predict HC in the general population and compare the predictive accuracy of five different ML algorithms based on data from the Korea National Health and Nutrition Examination Survey (KNHANES).

## 2. Methods

### 2.1. Study Population

This study was based on data from KNHANES 2008–2013, provided by the Korea Centers for Disease Control and Prevention. Using data from the KNHANES 2008–2013 databases, 10,089 subjects aged 12 years and older who had no missing responses on the questionnaire were included in this study. The KNHANES program was approved by the KNHANES Institutional Review Board (IRB) and was conducted in accordance with the Ethical Principles for Medical Research Involving Human Subjects, as defined by the Helsinki Declaration (IRB approval # 2013-12EXP-03-5C). All study participants provided informed written consent.

### 2.2. Data Collection

The KNHANES included well-established questions to determine the demographic characteristics and health status of the participants. This included questions on age, sex, income, physical exercise, smoking and drinking habits, and food intake. Daily energy intakes were assessed using 24 h recall and food intake frequency methods. The participants’ heights, weights and waist circumferences were measured. Then, the body mass index (BMI) was calculated as weight (in kilograms) divided by the square of height (in meters). Blood samples were collected by venipuncture after 10–12 h of fasting. Then, the total cholesterol was measured by enzymatic methods using commercially available kits (Sekisui Medical, Tokyo, Japan) within 2 h of blood sampling. The criterion for HC was a total cholesterol level of 240 mg/dL or higher or the use of lipid-lowering medications [22]. Furthermore, blood lead and cadmium were quantified using Zeeman effect graphite furnace atomic absorption spectrophotometry (Perkin-Elmer AAnalyst 600, Turku, Finland). Blood mercury was measured by cold-vapor atomic absorption spectrometry using a dedicated mercury analyzer (M-6000A; CETAC Technologies, Omaha, NE, USA). Details of the metal analysis have been reported elsewhere [23]. All blood metal analyses were carried out by a laboratory certified by the Korean Ministry of Health and Welfare.

### 2.3. Constructing the Data Sets and the Algorithm

All features were extracted from the original dataset and were transformed by scaling each feature to a range between zero and one using the ‘MinMaxScalar’ class from the pre-processing module of the Python scikit-learn library. After data processing, the input features were sex, age, income, BMI, waist circumference, exercise, smoking habits, alcohol drinking, and energy intake, as well as the blood concentrations of lead, mercury, and cadmium. Then, the entire data set was split into training and test sets at a ratio of approximately 7:3. Therefore, 8795 subjects were placed into the training set, while 1294 subjects were placed in the test set. The overall accuracy of each model was evaluated using k-fold cross-validation with k = 10. We used the default hyperparameter configurations unless otherwise specified. The LR model used L2 regularization with a primal formulation. The primal formulation was used because there are more samples than features. The regularization strength was set to 0.1, and the model was trained for 100 iterations before convergence. We used the KNN classifier ‘KNeighborsClassifier’ from sklearn.neighbors and the number of neighbor points was set to 3. In this study, the DT model used Gini impurity to measure the quality of split. The minimum number of samples required to split a node was set to two, and the minimum samples per leaf was set to one. The RF model used ten separate DT estimators and the SVM model used a linear type as its kernel with the shrinking heuristic enabled. The model used a C value of one and probability estimates were enabled to plot a receiving operating characteristic (ROC) curve for the model. All of the models were implemented using the scikit-learn library (version 0.20.1).

### 2.4. Statistical Analyses

We calculated the frequency and, where appropriate, the percentage or mean and standard deviation of the demographic characteristics to describe the sample population with respect to the HC categories. We compared the values of continuous variables between the HC and control (non-HC) groups using *t*-tests. The statistical significance of categorical variables between the HC and control groups were determined using the Mantel–Haenszel chi-square test. The prevalence of HC was compared among tertiles of blood metal concentration using multivariate logistic regression after adjusting for the other metals. As a measurement of the performance of the prediction model, the accuracy was calculated for all data, the training set, and the test set. The ROC curve was also used as a metric to measure the prediction model performance. All statistical analyses were carried out using SAS v. 9.4 (SAS Institute Inc., Cary, NC, USA).

## 3. Results

Table 1 shows the demographic characteristics and metal concentrations of the study population. Of the 10,089 participants included in the study, 46.4% were male and the mean age was 44.9 years. The blood concentrations of lead, mercury, and cadmium were 2.32 μg/dL, 4.55 μg/L, and 1.11 μg/L, respectively. Among the study participants, the prevalence of HC was 12.8%. Compared with the non-HC group, the HC group had significantly higher mean blood concentrations of lead, mercury, and cadmium, as well as mean values of age, BMI, and waist circumference (*p* < 0.01). The non-HC group had significantly higher mean monthly incomes, percentage of regular exercise, and mean daily energy intakes than the HC group (*p* < 0.01).

Table 2 shows the odds ratios (ORs) for the association of HC with blood metal levels. The trend in adjusted ORs for HC were significantly related to increased blood metal concentration. After adjustment for the other metals, the OR for HC was significantly correlated with blood levels of lead (*p* for trend = 0.001), mercury (*p* for trend = 0.020), and cadmium (*p* for trend < 0.001).

**Figure 1:**
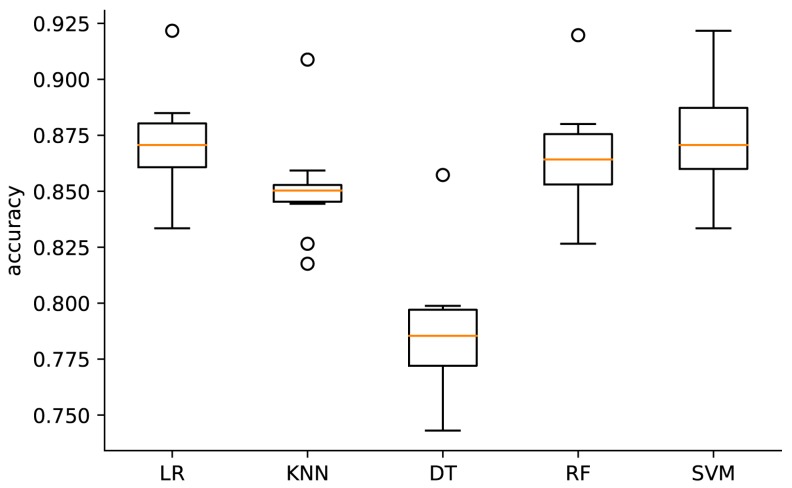
Box plot of the accuracy scores across each algorithm for normalized data. LR—logistic regression; KNN—k-nearest neighbor; DT—decision trees; RF—random forests; SVM—support vector machines.

The accuracies of all of the models are shown in Figure 1. Following the normalization of all features, the SVM model achieved the largest accuracy value (0.872) of all of the models, followed by the LR, RF, KNN, and DT models. There were no significant differences between the SVM model and LR prediction model (*p* = 0.174). However, the *p* values between the SVM or LR model, and all other models were less than 0.003.

Similarly, following the training of each model with the training dataset, the SVM and LR models had the best accuracy when analyzing the test dataset. On the other hand, the DT model has the lowest accuracy compared to other models (Table 3).

**Figure 2:**
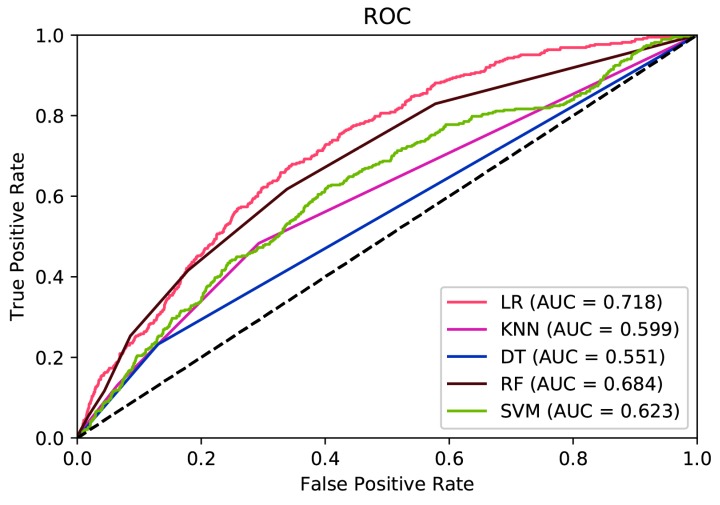
Receiver operating characteristic (ROC) curve of LR, KNN, DT, RF, and SVM models. LR—logistic regression; KNN—k-nearest neighbor; DT—decision trees; RF—random forests; SVM—support vector machines.

The area under ROC curve (AUC) for all of the prediction models is summarized in Figure 2. The LR model had the highest area under ROC curve of all of the prediction models. The LR model (AUC = 0.718, 95% CI = 0.688–0.748) was significantly different to all of the other prediction models except the RF model (AUC = 0.684, 95% CI = 0.652–0.714) using the standard 95% confidence interval criteria.

## 4. Discussion

Accumulating epidemiological and experimental studies have provided strong evidence that lead, mercury, or cadmium exposure can affect lipid metabolism, including the disturbance of total cholesterol levels [24,25,26]. Recently, based on the US National Health and Nutrition Examination Survey (NHANES) 2009–2012, higher levels of lead, mercury, and cadmium detected in the blood were reported to be associated with increased levels of total cholesterol [27]. This study was carried out to investigate the relative performance of various ML classification methods for predicting HC associated with exposure to metals. To the best of our knowledge, this is the first study to compare the performance of various ML models in terms of predicting HC using data from a nationally representative survey.

In the current study, we presented an empirical comparison of five different techniques for estimating the risk of HC using KNHANES data on 10,089 participants. The LR, RF, and SVM outperformed the DT and KNN techniques, achieving overall accuracies of >0.86 and area under the AUC of >0.60. The results of our study show that complex ML models, such as SVM, tend to outperform simpler models such as DT when predicting HC associated with exposure to heavy metals. As KNN is a non-linear classifier, it tends to perform better with many data points. Therefore, effective feature selection may improve the performance of the KNN classifier [28].

The strength of this study is that it is based on a large dataset, reflected by the number of participants and the number of important confounding variables, including cigarette smoking, alcohol drinking, physical exercise, and energy intake. Limitations of our study include the lack of more features and lack of further parameter optimization to avoid overfitting. In addition, clinical data, such as history of medication and prevalence of diseases affecting the lipid metabolism, were not included in the models, which may decrease the prediction accuracy of some of the algorithms. Nevertheless, the results of this study suggest that the performance of ML models can be very sensitive to the values of the hyperparameters and selected features. Therefore, the hyperparameters and features used to build ML models should be carefully explored and tuned to achieve the best predictive accuracy.

## 5. Conclusions

Prediction models using ML algorithms have shown solid prediction capabilities in various application domains including public health and environmental science. In this study, we presented a development and comparison of five popular ML models on predicating HC using a population-based database from the KNHANES. The results of this study showed that prevalence of HC among subjects exposed to lead, mercury, and cadmium could be predicted using different ML approaches with high accuracy. The results also show that various ML techniques can significantly vary in terms of their performance (accuracy and AUC) and that the SVM and LR models were the better models for predicting the risk of HC in the general population following exposure to lead, mercury, and cadmium. These findings suggest that ML approaches could be used as alternative methods in the prediction of the risk of HC due to exposure to metals.

## Figures and Tables

**Table 1:** Demographic characteristics by categories of hypercholesterolemia (HC).

Characteristic	Total (*n* = 10,089)	Non-HC (*n* = 8795)	HC (*n* = 1294)	*p* ^a^
Sex, male (%)	4681 (46.4)	4162 (47.3)	519 (40.1)	<0.001
Mean age (SD)	44.9 (15.6)	43.5 (15.6)	54.4 (12.4)	<0.001
Income, US$/month (SD)	2964 (2127)	2997 (2112)	2720 (2194)	<0.001
BMI, kg/m^2^ (SD)	23.6 (3.4)	23.4 (3.4)	24.9 (3.3)	<0.001
Waist circumference, cm (SD)	80.5 (10.0)	79.9 (9.9)	84.9 (9.2)	<0.001
Regular exercise (%)	2344(23.2)	2092 (23.8)	252 (19.5)	0.001
Energy intake, kcal/day (SD)	2015.2 (881.8)	2030.3 (887.4)	1912.9 (835.5)	<0.001
Lead, μg/dL (SD)	2.32 (1.20)	2.30 (1.22)	2.45 (1.02)	<0.001
Mercury, μg/L (SD)	4.55 (3.90)	4.49 (3.55)	4.94 (5.71)	0.006
Cadmium, μg/L (SD)	1.11 (0.68)	1.08 (0.68)	1.29 (0.67)	<0.001

^a^*p*—determined by *t*-test or Mantel–Haenszel chi-square test between HC and no-HC groups. HC—hypercholesterolemia; BMI—body mass index.

**Table 2:** Adjusted odds ratios and 95% confidence intervals of hypercholesterolemia by blood metal level.

Metal ^a^	Tertile Blood Metal Level			*p* for Trend ^b^
	Low (*n* = 3363)	Middle (*n* = 3363)	High (*n* = 3363)	
Lead	1.00 (reference)	1.06 (0.92–1.21)	1.29 (1.10–1.51)	0.006
Mercury	1.00 (reference)	1.22 (1.06–1.41)	1.15 (0.99–1.33)	0.020
Cadmium	1.00 (reference)	1.36 (1.19–1.56)	2.31 (1.97–2.71)	<0.001

^a^ Adjusted for the other metals in the table; ^b^ *p*—determined by linear contrast in logistic regression model.

**Table 3:** Accuracy values for all prediction models.

Dataset	LR	KNN	DT	RF	SVM
All	0.870	0.851	0.787	0.865	0.872
Train	0.872	0.899	1.000	0.981	0.872
Test	0.872	0.832	0.788	0.864	0.872

LR—logistic regression; KNN—k-nearest neighbor; DT—decision trees; RF—random forests; SVM—support vector machines.
